# Observation of fractional spin textures in a Heusler material

**DOI:** 10.1038/s41467-022-29991-1

**Published:** 2022-04-29

**Authors:** Jagannath Jena, Börge Göbel, Tomoki Hirosawa, Sebastián A. Díaz, Daniel Wolf, Taichi Hinokihara, Vivek Kumar, Ingrid Mertig, Claudia Felser, Axel Lubk, Daniel Loss, Stuart S. P. Parkin

**Affiliations:** 1 Max Planck Institute of Microstructure Physics, Weinberg 2, 06120 Halle, Germany; 2 Institute of Physics, Martin Luther University Halle-Wittenberg, 06120 Halle, Germany; 3 Department of Physics, University of Tokyo, Bunkyo, Tokyo 113-0033 Japan; 4 Department of Physics, University of Basel, Klingelberg Strasse 82, 4056 Basel, Switzerland; 5 Faculty of Physics, University of Duisburg-Essen, 47057 Duisburg, Germany; 6 Institute for Solid State Research, IFW Dresden, Helmholtzstrasse 20, 01069 Dresden, Germany; 7 Elements Strategy Initiative Center for Magnetic Materials, National Institute for Materials Science, Tsukuba, Ibaraki 305-0047 Japan; 8 Max Planck Institute for Chemical Physics of Solids, Nöthnitzer Strasse 40, 01187 Dresden, Germany

**Keywords:** Spintronics, Magnetic properties and materials

## Abstract

Recently a zoology of non-collinear chiral spin textures has been discovered, most of which, such as skyrmions and antiskyrmions, have integer topological charges. Here we report the experimental real-space observation of the formation and stability of fractional antiskyrmions and fractional elliptical skyrmions in a Heusler material. These fractional objects appear, over a wide range of temperature and magnetic field, at the edges of a sample, whose interior is occupied by an array of nano-objects with integer topological charges, in agreement with our simulations. We explore the evolution of these objects in the presence of magnetic fields and show their interconversion to objects with integer topological charges. This means the topological charge can be varied continuously. These fractional spin textures are not just another type of skyrmion, but are essentially a new state of matter that emerges and lives only at the boundary of a magnetic system. The coexistence of both integer and fractionally charged spin textures in the same material makes the Heusler family of compounds unique for the manipulation of the real-space topology of spin textures and thus an exciting platform for spintronic and magnonic applications.

## Abstract (web summary)

Skyrmions and anti-skyrmions are magnetic textures that have garnered much interest due to their stability. Here, Jena et al demonstrate the existence of fractional spin textures at the edges of Heusler alloy sample, which can have continuous variable topological charges.

## Introduction

The mathematical concept of topology has proven to be highly relevant in several fields of natural sciences^1–4^. In condensed matter physics, it is used to classify and explain the enhanced stability of distinct phases, and as a guiding principle for the design of materials and systems with novel, robust properties. When infinitely extended periodic systems exhibit non-trivial reciprocal-space topology, characterized by integer-valued topological invariants computed from the band structure of their collective excitations, topologically protected boundary states emerge^5–8^–the hallmark of the bulk-boundary correspondence^9,10^. In magnetism, non-collinear spin textures can also be characterized by a topological invariant, albeit defined in real space. This ‘topological charge’ or ‘skyrmion number’ *N*_sk_ is ±1 for most of the recently observed objects^11,12^, including skyrmions and antiskyrmions, which are mesoscopic magnetic whirls that have been observed in chiral systems^13–15^. An integer value of *N*_sk_ is guaranteed under the assumption of a continuous spin texture and that the surface on which it resides (the magnetic unit cell or the whole sample depending on the periodicity of the texture) can be mapped to a sphere. As a counter-example, magnetic whirls called merons, stabilized in in-plane magnetized systems^16^, carry *N*_sk_ = ±1/2 as their sample surface cannot be mapped to a sphere. Objects with fractional topological charge have been realized in the interior of the sample, but so far, they always maintain a net integer *N*_sk_ in the unit cell^17–19^, making them topologically equivalent to conventional skyrmionics excitations.

Under the assumption of a continuous spin density, as in the non-linear sigma model, skyrmions and antiskyrmions are topologically protected by an infinite energy barrier from transforming into a trivial state, with *N*_sk_ = 0, such as a collinear ferromagnet. In fact, since this model describes the limit where the lattice constant goes to zero, a discontinuous change in the topological charge can only occur via the introduction of a Bloch point singularity^20^. For this reason, it is commonly believed that the above mentioned non-collinear spin textures are stable due to their non-trivial real-space topology. However, real spin textures are defined on a lattice. Still, a discretized, integer-valued version of *N*_sk_ can be defined^21^ and the now *finite* energy barriers between different topological sectors can be overcome. This is even more prominent in a confined geometry, where the continuum approximation breaks down at edges of a sample. As we prove here, by observing fractionally topologically charged objects that are not allowed in a continuum model, the energy landscape, determined by the magnetic interactions, must be more important than the topological properties of spin textures. A route that allows for non-integer topological charges is the introduction of an edge.

**Fig. 1:**
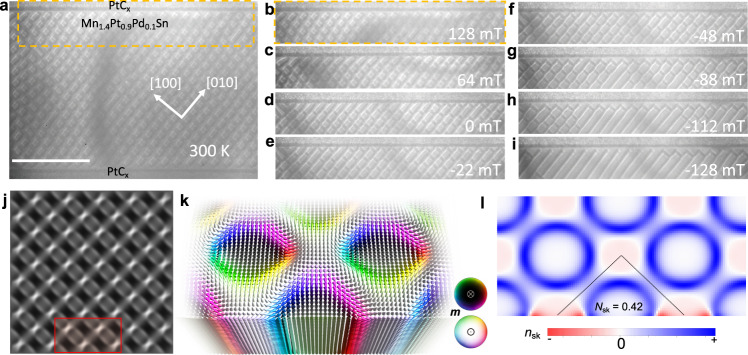
Formation of fractional antiskyrmions at an interface of Mn_1.4_Pt_0.9_Pd_0.1_Sn. **a** The lamella is filled with a dense lattice of antiskyrmions and a few short helices at 128 mT and 300 K. The region marked by the dashed orange rectangle in (**a**) is shown for various magnetic fields in (**b**–**i**). Note that (**b**) is the same image as in (**a**). **c** Image in a field of 64 mT. **d** Fractional antiskyrmions that form at the interface region with PtC_x_ are elongated in the absence of a magnetic field. **e**–**f** When a negative magnetic field is applied, the fractional antiskyrmions become smaller in size. **h**, **i** At fields beyond −100 mT, the antiskyrmions in the interior region first join with each other and form helices and then these helices join with the fractional objects so that a helical state is also found at the interface. The scale bar in (**a**) corresponds to 2 μm. **j** Simulated LTEM contrast of an antiskyrmion lattice with fractional antiskyrmions at the edge that was stabilized in micromagnetic simulations. **k** A magnified view of the magnetic texture of the red area in (**j**) with arrows and colors indicating the orientations of the magnetic moments. **l** The topological charge density of the spin texture in (**k**).

**Fig. 2:**
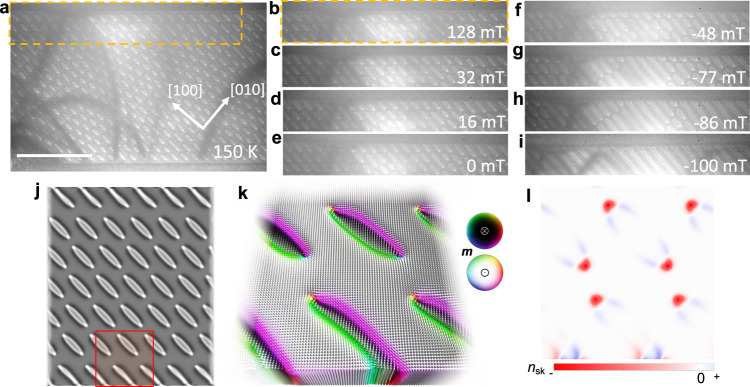
Formation of fractional elliptical Bloch skyrmions. **a** A lattice of elliptical Bloch skyrmions at 128 mT and 150 K. **b** The region identified by the dashed orange rectangle in (**a**) is shown in (**b**–**i**). **c** At 32 mT, fractional Bloch skyrmions start to form at the interface with PtC_x_. **d**, **e** The number of fractional nano-objects increases when the elliptical Bloch skyrmions enlarge and join the interface. **f** In negative fields, the size of the elliptical skyrmions can decrease because the bulk elliptical skyrmions enlarge. **g**, **h** Upon further increasing the negative field, the fractional nano-objects start to merge with the bulk elliptical Bloch skyrmions and form helices. **i** A helical state is found everywhere including the interface region. The scale bar in (**a**) corresponds to 2 μm. **j** Simulated LTEM contrast of an elliptical skyrmion lattice with fractional objects at the top and bottom edges that was stabilized in micromagnetic simulations. Periodic boundary conditions have been applied along the horizontal direction. **k** A magnified view of the magnetic texture of the red area with arrows and colors indicating the orientation of the magnetic moments. **l** The topological charge density of the spin texture in (**k**).

Herein, we consider skyrmions and antiskyrmions that carry a fractional topological charge and emerge along the edge of the sample. They are fundamentally different from topological edge defects in finite in-plane magnetized systems, whose winding number takes fractional values^22^. We experimentally observe the formation, stability, and annihilation of fractional antiskyrmions (Fig. 1) and fractional elliptical skyrmions (Fig. 2) at the edges of the Heusler material Mn_1.4_Pt_0.9_Pd_0.1_Sn. Our real space observations, conducted by Lorentz transmission electron microscopy (LTEM), are supported by micromagnetic simulations of the magnetic texture to reveal the stabilizing mechanism of these objects. Using atomistic simulations, we show that fractional skyrmions should also exist in typical skyrmion-hosting materials, like the B20 material MnSi, but their observation is difficult, due to a tilting of the fractional skyrmion tubes, and should be facilitated by alternative imaging techniques.

## Results and discussion

The LTEM images that we present in the following were acquired from a thin specimen prepared using a Ga^+^-ion dual beam focused ion beam method (Supplementary Fig. S1). More details of the preparation method can be found elsewhere^23^.

### Edge twist and the metastability of fractional objects

Before we present our LTEM measurements and simulations, we want to convey the general idea as to why fractional objects may emerge at sample edges. Non-collinear spin textures on a lattice are not stabilized by topology alone. Instead, magnetic interactions are responsible for their stability. In systems with broken inversion symmetry, the Dzyaloshinskii–Moriya interaction (DMI)^24,25^ is the most relevant. Depending on the type of symmetry breaking, the interaction can be isotropic, as in MnSi, stabilizing rotationally symmetric skyrmions^14^, or can be anisotropic, as in the Heusler material Mn_1.4_Pt_0.9_Pd_0.1_Sn, stabilizing antiskyrmions^15^.

Spins along the sample edge have missing neighbors leading to uncompensated DMI bonds that result in an edge twist of the texture^26,27^. The sample surface, hosting such an edge-twisted texture, can no longer be mapped to a sphere, thus the topological charge is not restricted to take on integer values: Even for a trivial ferromagnetic or helical state, the twisted edge leads to a small *N*_sk_ ≠ 0. Below a critical magnetic field, the twisted texture along the edge becomes unstable against the nucleation of incipient stripe domains^28^. In atomistic simulations of a confined geometry, the energy landscape clearly shows that a fractional antiskyrmion is energetically more favorable than a complete antiskyrmion at low magnetic fields, as simulated in Supplementary Fig. S3. Therefore, as our measurements will reveal, (anti)skyrmions from the interior that are sufficiently close to the edge can further reduce their energy by transforming into such incipient stripe domains. The nucleated domains, repelled by a crystal of (anti)skyrmions, stabilize along the edge as fractional (anti)skyrmions.

In Fig. 1k fractional antiskyrmions are shown, obtained by micromagnetic simulations (details and parameters can be found in the Methods section). While the twist in the region surrounding the fractional antiskyrmions is into the vacuum direction, inside the antiskyrmions the twist is along the opposite direction, i.e., into the interior of the sample. If fractional antiskyrmions were pushed toward the edge of the sample, the twist would have to be overcome. Therefore, this edge twist brings about an energy barrier that protects fractional antiskyrmions from spontaneous annihilation. Pushing fractional objects in the opposite direction would not be hindered by the edge twist, but by the repulsive interaction from the bulk antiskyrmions.

### Experimental observation of fractional antiskyrmions

We start by discussing the measured textures at room temperature. As was presented in our earlier study^23^, at this temperature antiskyrmions form in the interior of the lamella. However, in these thin lamellae of the *D*_2d_ Heusler compound, a special protocol has to be used in order to stabilize them. If we simply apply an out-of-plane magnetic field, only a few antiskyrmions form and the phase diagram is dominated by the helical and the ferromagnetic phases. However, when an in-plane field is provided temporarily by reversibly tilting the sample in the TEM column by ~40°, the nucleation of antiskyrmions is triggered. We start from a large field, so that the ferromagnetic phase is stabilized. Next, we reduce the field to a value B^*^ and reversibly tilt the sample once to provide the in-plane field component. Thereafter, the perpendicular magnetic field is reduced without tilting the sample.

B^*^ serves as a parameter to control the antiskyrmion density in our sample. Depending on the magnitude of B^*^, a sparse or dense array of antiskyrmions is formed^23^. In Fig. 1, we start from a rather dense array of antiskyrmions in the interior of the sample (B^*^ around 304 mT). Upon further decreasing the field to *B* = 128 mT, the antiskyrmion lattice remains stable; cf. Fig. 1a. The antiskyrmions have the same square-shaped contrast that we have analyzed in our previous study^23^. This deformation is a signature of the dipole-dipole interaction and the anisotropic DMI^29^. Due to these interactions, the square-shaped antiskyrmions form a square lattice. This antiskyrmion lattice reaches close to the edge, which is an interface between the magnetic Mn_1.4_Pt_0.9_Pd_0.1_Sn and the non-magnetic PtC_x_ applied during the fabrication of the lamella.

In the following, we concentrate on this region of the sample (orange border; shown also in Fig. 1b) and discuss the texture upon decreasing the magnetic field. In Fig. 1c the field is 64 mT and the size of the square-shaped antiskyrmions has increased. At 0 mT, the antiskyrmions that are closest to the edge have turned into fractional objects (Fig. 1d). Upon applying negative magnetic fields in Fig. 1e, f, the bulk antiskyrmions become more and more square-shaped because their size would increase if they were not confined by the neighboring antiskyrmions. This pushes the fractional antiskyrmions, which now appear as triangles, even closer to the edge: They are square-shaped antiskyrmions that have been ‘cut in half’. Upon further increasing the magnitude of the negative field, the fractional antiskyrmions merge among themselves forming a polarized region in the vicinity of the edge, while the bulk antiskyrmions also merge and turn more and more into helices; cf. Fig. 1g–i.

**Fig. 3:**
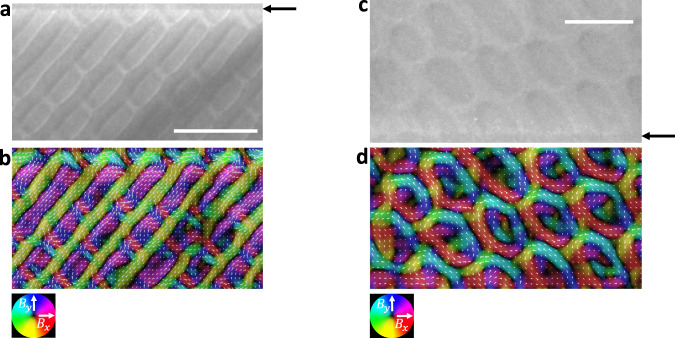
Transport of intensity equation (TIE) analysis of fractional (anti)skyrmions. **a** LTEM image at a magnetic field of −14.5 mT and a temperature of 200 K. The boundary between Mn_1.4_Pt_0.9_Pd_0.1_Sn and PtC_x_ is marked by black arrows. **b** The corresponding in-plane magnetic field reconstructed by solving the TIE. The color indicates the in-plane field direction according to the small white arrows. **c** LTEM image at −16 mT and 125 K. **d** The corresponding in-plane magnetization. Both LTEM images are taken at the defocus value of ∼0.3 mm. LTEM images at different defocus values are shown in supplement Figs. S8–9. The scale bars in (**a**), (**c**) correspond to 300 nm.

In Fig. 3b we present transport of intensity equation (TIE) reconstructions of the magnetic texture from the measured LTEM contrast (for details see the Methods section and SI). One can clearly see that the characteristic contrast of the bulk antiskyrmions are only partly present near the edge. Note, however, that the reconstruction of the full magnetic texture of antiskyrmions is difficult due to the Néel walls that deliver very small contrast in the LTEM measurements. Therefore, next we consider micromagnetic simulations of the texture and the corresponding simulated LTEM contrast.

In Fig. 1k we have stabilized a square lattice of square-shaped antiskyrmions with fractional antiskyrmions at the edge in the micromagnetic framework used (see Methods for details). This texture resembles the experimentally observed system qualitatively well. The corresponding simulated LTEM contrast is shown in Fig. 1j (see Methods for details). In Fig. 1k a magnified view of the red highlighted region is shown. The color encodes the orientation of the magnetic moments but here their orientation is also visualized by arrows. It becomes apparent that the antiskyrmions are practically cut in half at the edges but also that they slightly deform due to the edge twist. As discussed above, this twist constitutes an energy barrier enabling the stability of the fractional objects. Also, it leads to a redistribution of topological charge density that is shown in Fig. 1l. An antiskyrmion that is stabilized in a positively magnetized background has an exclusively positive topological charge density that integrates to almost 1 (here it is 0.99 for bulk antiskyrmions due to the discussed lattice effects). However, directly at the edge, negative contributions to the topological charge arise. Integrating over a fractional antiskyrmion yields the topological charge 0.42 which is quite far from 0.5 expected without the edge twist. This value depends on the magnetic interaction parameters, as well as the size of these objects.

As mentioned above, we can tune the density of the bulk antiskyrmion lattice by the field B^*^. For an intermediate density of bulk antiskyrmions, fractional antiskyrmions form as well but some of them elongate far into the interior of the sample (Fig. S4). As discussed before, the edge tilting only protects the fractional antiskyrmions from annihilation but not from extension into the interior. This extension can only be suppressed by the repulsive interaction with bulk antiskyrmions. For a low density of bulk antiskyrmions, no fractional antiskyrmions have formed. Consequently, the bulk antiskyrmions are essential for the formation of these objects at the edges. This becomes also apparent when we compare the presented results with our recent study on (anti)skyrmions in nanostripes^30^. In that paper the sample had a width less than 500 nm and could host only two rows of antiskyrmions. Under no circumstances did we observe fractional objects in that sample. Apparently, this is because no real bulk antiskyrmion lattice can form in such a geometry. When we start from an antiskyrmion lattice and field-cool the sample to low temperatures, the antiskyrmion lattice and the fractional antiskyrmions survive, as is shown in Supplementary Fig. S5.

It is to be noted that the role of PtC_x_ is to protect the sample during the sample processing and to produce an interface region that is free from Fresnel fringes, which negatively affect LTEM measurements. However, adding PtC_x_ is not mandatory for the formation of fractional nano-objects. In Supplementary Fig. S6 we show that fractional antiskyrmions also form at the interface of Mn_1.4_Pt_0.9_Pd_0.1_Sn with vacuum even though this interface is rougher due to the preparation process.

### Experimental observation of fractional elliptical Bloch skyrmions

At low temperatures the antiskyrmion is not the naturally stable type of texture. The dipole-dipole interaction, which is the dominating interaction at low temperatures, leads to the stabilization of Bloch skyrmions instead of antiskyrmions, as explained in ref. ^23,31^. Due to the anisotropic DMI, these skyrmions are elongated along the [100] direction for clock-wise Bloch skyrmions that we find here, stabilized under the same experimental protocol as before but at lower *T*. Due to this elongation, we have dubbed them ‘elliptical skyrmions’. In Fig. 2, it is shown that the stabilization of these objects at 150 K is very similar to the antiskyrmionic case. Again, we can control the density of non-collinear objects by tuning B^*^ as shown in Supplementary Fig. S7. This time the dense starting lattice of elliptical skyrmions that was created by B^*^ near 384 mT remains stable also at 128 mT in Fig. 2b. Again, upon decreasing the perpendicularly applied magnetic field, these objects become increasingly larger until the skyrmions closest to the edge touch the edge and form fractional objects. Since these skyrmions do not form a square-shaped lattice but rather a hexagonal lattice, we have considered periodic boundary conditions along the horizontal direction in the simulations. This avoids finite-size related quenching effects of the skyrmions due to the square-shaped geometry. At the top and the bottom of that simulated spin cluster, we find fractional elliptical skyrmions (Fig. 2j). Looking at the texture of such an object in detail, cf. Fig. 2k, we can again see the edge twist.

In Fig. 3d, we present TIE reconstructions of the magnetic texture based on the measured LTEM contrast. The TIE reconstructions agree well with the simulated textures and clearly show the existence of fractional Bloch skyrmions near the edge. Note that the projected magnetic induction map shown in Fig. 3d that is computed by TIE from the L-TEM image in Fig. 3c amplifies slight contrast variations in the L-TEM image, which are otherwise barely visible. This leads to some fine features inside the skyrmion textures in the TIE image such as a reversal of helicity. However, these details are close to the detection limit of the LTEM method.

### Conversion vs. annihilation mechanism

**Fig. 4:**
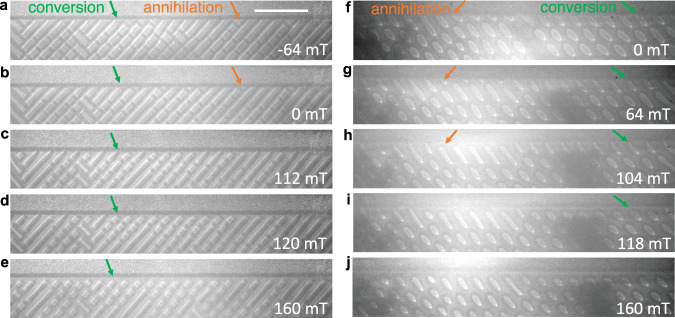
Conversion mechanisms of fractional nano-objects. **a** Fractional antiskyrmions of various sizes are observed at −64 mT and 300 K. The magnetic field is increased from this state and the corresponding LTEM images are shown in (**b**–**e**). **b** The fractional antiskyrmions that are shorter decrease in size. **c** At 112 mT, the short fractional nano-objects (e.g., marked by the orange arrow) vanish and the longer fractional nano-objects (e.g., marked by the green arrow) are pinched off at the edge. **d**, **e** Upon increasing the magnetic field, the longer fractional antiskyrmions have transformed to bulk antiskyrmions. No fractional antiskyrmions are present. Similar mechanisms are observed for fractional elliptical Bloch skyrmions at 150 K as illustrated in **f**–**j**. The zero-field state in (**f**) was obtained by the procedure given in Fig. 2. Depending on their initial size, the fractional elliptical Bloch skyrmions can annihilate (orange arrow) or can form complete elliptical skyrmions in the bulk (green arrow). The scale bar in (**a**) corresponds to 1 μm.

Above, we have discussed the decreasing field behavior of the magnetic textures. Next, we present how fractional antiskyrmions and fractional elliptical skyrmions evolve when the field is increased. In both cases we start from a dense lattice of bulk (anti)skyrmions with fractional objects at the edge at −64 mT and 0 mT in Fig. 4a, f, respectively. When the field is increased, the size of the bulk objects shrinks. This brings about two counter-acting effects for the fractional (anti)skyrmions. The increased field favors a shrinking size of the fractional objects per se but since the bulk objects also shrink, their repulsive interaction decreases, which allows for an elongation of the fractional (anti)skyrmion. The dominating behavior of these two counter-acting trends depends strongly on the starting configuration. If we look at a small fractional skyrmion or antiskyrmion (red arrow in Fig. 4a, f), this object shrinks until it disappears. On the other hand, if we focus on a fractional object that is initially elongated, due to a lower density of bulk objects in its vicinity, the fractional (anti)skyrmion elongates, and finally nucleates a new bulk (anti) skyrmion (green arrow in Fig. 4a, f). The conversion-annihilation mechanism is further illuminated by performing atomistic spin dynamics simulations (see Supplementary Video 1). Furthermore, the merging process of fractional antiskyrmions at negative magnetic fields was simulated in Supplementary Video 2.

For both objects and for both transformation mechanisms—annihilation and conversion—the topological charge density varies continuously. No fractional objects remain stable at increased fields. This points to the fact that bulk (anti)skyrmions are more stable than fractional (anti)skyrmions and that the latter disappear or transform before the bulk objects are affected. Since the number of topological objects has to decrease when the magnetic field is increased, the fractional objects do not remain stable at higher fields. This observation is in accordance with our prior observation that fractional (anti)skyrmions do not exist individually but always require a rather dense interior region for their formation in the decreasing field mode.

### Comparison to B20 materials

In this paper, we have discovered, for the first time, the existence of fractional skyrmions and antiskyrmions. Note that a single fractional bubble can be seen in one of the figures in ref. ^32^, but there was no discussion of this object. The question arises why these objects have not yet been seen in typical skyrmion hosts, like the B20 material MnSi, despite the fact that these materials have been investigated far more extensively than the antiskyrmion-hosting Heuslers.

**Fig. 5:**
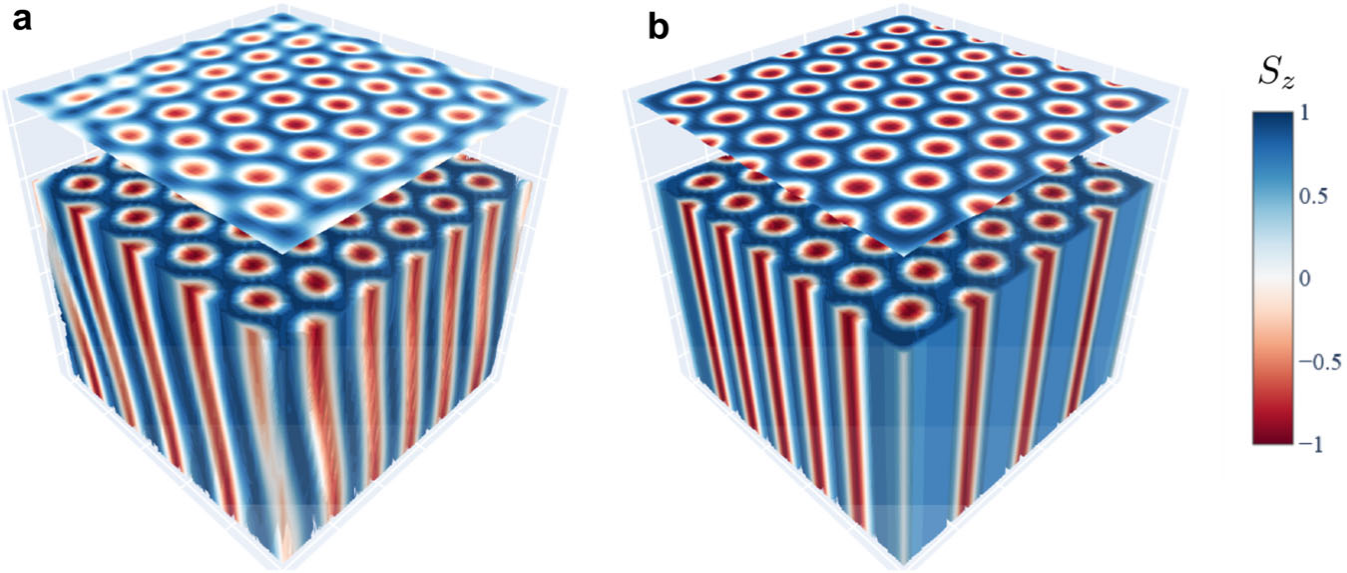
Three-dimensional profile of boundary fractional (anti)skyrmions. Three-dimensional magnetic configuration of confined skyrmion lattices obtained by atomistic spin Monte Carlo simulations with (**a**) bulk DMI, characteristic of B20 materials, and (**b**) with *D*_2d_ DMI, typical of Heuslers. The average of the out-of-plane spin components over the sample thickness is displayed atop the skyrmion lattices. Skyrmionic objects exhibit a vertical tubular structure in Heuslers allowing their clear observation via imaging techniques such as LTEM (**b**). The DMI in B20 materials, however, favors tilted skyrmionic tubes resulting in blurry images along the sample edge (**a**). This could explain why fractional skyrmions have not been detected in B20 materials by LTEM or similar imaging techniques. The dipolar interaction was not included. In the presence of the dipolar interaction, an equivalent result is obtained with a less pronounced tilting of the tubes.

Following from our atomistic simulations presented in Fig. 5, fractional Bloch skyrmions can, in principle, also be stabilized in materials with an isotropic DMI like in MnSi. However, one also has to take into account the full three-dimensional texture to understand the difference between both material classes. The anisotropic DMI in Heusler materials is a two-dimensional interaction that acts layer-wise. Instead, the bulk DMI in B20 materials also has DMI vectors for bonds along the *z* direction. This brings about two consequences. On the one hand, it leads to the emergence of a conical phase in B20 materials that is in conflict with the skyrmionic phase and consequently reduces its stability in the phase diagram. This phase is absent for antiskyrmions in Heusler materials, leading to an enhanced stability and a larger region in the phase diagram^15,33^. In B20 materials, the skyrmion lattice, required for the stability of fractional skyrmions, may itself no longer be stable in the low magnetic field range where the latter can emerge.

On the other hand, even if fractional skyrmions were stable in B20 materials, the tubes of fractional Bloch skyrmions in B20 materials are tilted while the tubes of fractional antiskyrmions in Heusler materials are straight. Like in the present study, typically, the real-space texture is experimentally measured by techniques that average the signal over the thickness of the sample. If we observe a straight tube, for which the magnetization does not change significantly throughout the layers, this is unproblematic: the measured signal can be assumed to represent the magnetic texture of every single layer. However, for the tilted tubes in B20 systems this becomes problematic: In Fig. 5 we also show the averaged magnetization over all layers. While the bulk Bloch skyrmions can be observed without a problem, as has been done in many publications, the fractional skyrmions at the edge result in a smeared-out signal due to the tilted tubes. This signal is very similar to a signal that arises from a mere edge twist without the emergence of fractional objects as was assumed in FeGe^34^. In summary, fractional Bloch skyrmions can exist in B20 materials and could be observed by imaging techniques that average over the sample thickness as long as the thickness is comparable to the size of skyrmions. On the other hand, for thicker samples, three-dimensional imaging techniques should be utilized such as X-ray nanotomography^35^ or electron holography^36^.

We have presented the experimental discovery of the formation of fractional antiskyrmions and fractional elliptical skyrmions in a material with an anisotropic DMI. These objects are stable in a finite field and temperature range only when a considerably dense bulk (anti)skyrmion lattice is present. Fractional objects cannot exist on their own. While the bulk lattice hinders an elongation of the fractional (anti)skyrmions, the DMI-mediated edge twist protects them from spontaneous annihilation via the edge. By tuning the field, we can continuously control their topological charges because they either annihilate or convert to integer-charged objects. Furthermore, we have predicted by simulations that fractional objects are not unique to materials with an anisotropic DMI like Heuslers. Fractional Bloch skyrmions can exist in materials with a three-dimensional DMI like the B20 material MnSi as well. However, it is hardly possible to observe them by techniques that integrate the signal over the whole thickness of the sample.

Our observation of fractional (anti)skyrmions proves that magnetic interactions are mostly relevant for the stability of non-collinear spin textures at the boundaries instead of formal topological protection that has its origin in continuum models. Our findings bring about new possibilities for realizing fascinating fundamental effects and advantageous spintronic and magnonic applications. This includes the realization of the recently predicted magnonic quadrupole topological insulator, supported by (anti)skyrmion lattices^37^, in which fractional (anti)skyrmions restore the protecting symmetries that guarantee the emergence of robust magnonic corner states, hallmarks of the bulk-boundary correspondence of this higher-order topological phase. Boundary fractional (anti)skyrmion tubes could support magnonic modes similar to those recently reported in skyrmion tubes^38,39^, thus they could act as robust, reconfigurable magnonic waveguides running along the lateral facets of the sample. The demonstrated topological charge annihilation and conversion processes identify Heuslers as unique platforms to investigate the emergent electrodynamics^40^ arising from spin textures with continuously tunable topological charge. Furthermore, our simulations reveal that fractional topological charges are a general concept that is to be expected also for other members of the skyrmionic family.

## Methods

### Experiment

The bulk polycrystalline Heusler compound Mn_1.4_Pt_0.9_Pd_0.1_Sn was prepared by an arc-melting method, as described elsewhere^15^. The electron backscattering diffraction technique was performed in a TESCAN GAIA 3 (Quantax, Bruker) system to realize [001]-oriented grains. The single crystalline lamellae with parallel surfaces were fabricated from single [001] oriented grains by a Ga^+^ ion-based dual beam focused ion beam system [FEI Nova Nanolab 600 SEM/FIB] operating at an accelerating voltage of 30 kV. Low energy polishing at 5 kV and then at 2 kV were performed to remove any damaged surface material. An aberration-corrected high-resolution transmission electron microscope [FEI TITAN 800-300] was used to observe the magnetic contrast in the LTEM mode. The magnetic field was varied from −0.28 T to 2.3 T along the microscope axis by partially exciting the objective lens. The magnetic field values were calibrated by a Hall bar sensor. The double tilt liquid nitrogen holder was used to vary the temperature of the specimen. The in-plane field components were realized at the specimen surface by tilting the sample holder up to ±40°. The under-focus value for the LTEM imaging is ~0.9 mm.

### Micromagnetic simulations

For the micromagnetic simulations we started from an analytically constructed seed and propagated the magnetic texture toward the nearest energy minimum using mumax3^41,42^. The propagation is according to the Landau–Lifshitz–Gilbert equation^43^ (Eq. 1)

1 $$\dot{{{{{{\bf{m}}}}}}}=-{\gamma }_{e}{{{{{\bf{m}}}}}\,}\times {{{{{{\bf{B}}}}}}}_{{{{{{\rm{eff}}}}}}}+\alpha {{{{{\bf{m}}}}}\,}\times \dot{{{{{{\bf{m}}}}}}}$$

where ${{{{{{\bf{B}}}}}}}_{{{{{{\rm{eff}}}}}}}=\frac{-\delta F}{{M}_{{{{{{\rm{S}}}}}}}\delta {{{{{\bf{m}}}}}}}$ is the effective magnetic field that is computed from the free energy *F*. This energy includes the exchange interaction *A*, anisotropic DMI *D*, an easy-axis anisotropy *K*, interaction with a magnetic field **B** and dipolar interactions. The parameters have been taken from our recent publication on nanostripes of Mn_1.4_Pt_0.9_Pd_0.1_Sn^30^: *D* = 0.003 J/m^2^, *A* = 0.5 × 10^−10 ^J/m, *K* = 135 kJ/m^3^, Gilbert damping *α* = 0.3, saturation magnetization *M*_S_ = 445 kA/m. These parameters allowed for the metastability of fractional antiskyrmions, as shown in Fig. 1 and Supplementary Fig. S4, in a finite geometry of size 162 nm × 162 nm × 280 nm with a cell size 5 nm × 5 nm × 10 nm. For the fractional skyrmions in Fig. 2 we had to account for an anisotropy gradient close to the edge due to the PtC_x_: we used a 50% increased *K* in the bulk region. Note that this is only required for the present parameters and is not generally needed as our atomistic simulations establish. Furthermore, we used periodic boundary conditions along the *x* direction to reduce finite-size effects. We simulated a region 240 nm × 290 nm × 280 nm. While antiskyrmions form a square lattice compatible with a square-shaped geometry, the skyrmions form a hexagonal lattice. We used a magnetic field of 0 mT in Fig. 1 and Supplementary Fig. S4, and 180 mT in Fig. 2.

### Lorentz TEM simulations

We have simulated the Lorentz TEM images of the (anti)skyrmionic spin structure obtained from micromagnetic simulations. To this end, we calculated from the latter the 3D magnetic vector potential **A** (in Coulomb gauge) and projected its *z*-component along the electron beam direction *z* to finally compute the Aharonov–Bohm phase shift of the transmitted electron wave function. Finally, this wave function was convoluted with the defocus-dependent point-spread function (i.e., by multiplying the corresponding aberration function in Fourier space).

### Transport of intensity equation (TIE) analyses

Transport of intensity phase reconstruction was performed by acquiring two mutually slightly defocused images, one exactly in focus showing no magnetic contrast and one at 0.3 mm over-focus *D*_*z*_, showing weak magnetic contrast above the noise level. The images were then aligned with respect to each other via autocorrelation. Subsequently, the field of view has been cropped to contain only the Mn_1.4_Pt_0.9_Pd_0.1_Sn part of the FIB lamella, showing no amplitude contrast in focus except the unavoidable bending contours. Following these preprocessing steps TIE reconstruction corresponding to solving the elliptic partial differential equation (Eq. 2)

2 $$\frac{I\left(x,y,{D}_{z}\right)-I\left(x,y,0\right)}{{D}_{z}}\approx -\frac{1}{{k}_{0}}\nabla \,\cdot\, I\left(x,y\right)\nabla {{{{{\rm{\varphi }}}}}}\left(x,y\right)$$

(*k*_0_ is the wave number of the electron beam) with respect to the phase φ, employing von-Neumann boundary conditions, has been carried out with the help of a Fourier space solver. In order to suppress ubiquitous reconstruction error amplification of the mildly ill-conditioned TIE reconstruction at small spatial frequencies, an adapted high-pass filter suppressing small spatial frequencies around the origin of reciprocal space has been incorporated in the reconstruction procedure. Following the phase reconstruction, the projected in-plane magnetic fields were obtained from

3 $${\int }_{0}^{t}{({B}_{x},{B}_{y})}^{T}dz=\frac{\hslash }{e}{(-{\partial }_{y}{{{{{\rm{\varphi }}}}}},{\partial }_{x}{{{{{\rm{\varphi }}}}}})}^{T}$$

(sample thickness *t*) (Eq. 3). Notwithstanding, reconstruction artifacts at the boundary of the reconstructed field of view (containing the crucial interface region) could not be completely suppressed (see, e.g., right interfacial region in Fig. S10), which mainly originate from violation of assumed von-Neumann boundary conditions and non-magnetic contrast variations in the original images.

### Atomistic spin simulations

We use a cubic spin lattice model with the following Hamiltonian (Eq. 4)

4 $${{{H}}}=	\frac{{{{1}}}}{{{{2}}}}\mathop{\sum}\limits _{{{{{{\bf{r}}}}}},{{{{{{\bf{r}}}}}}}^{{{{\prime} }}}}\left(-{{{{J}}}}_{{{{{{\bf{r}}}}}},{{{{{{\bf{r}}}}}}}^{{\prime} }}{{{{{{\bf{S}}}}}}}_{{{{{{\bf{r}}}}}}}\cdot {{{{{{\bf{S}}}}}}}_{{{{{{{\bf{r}}}}}}}^{{\prime} }}+{{{{{{\bf{D}}}}}}}_{{{{{{\bf{r}}}}}},{{{{{{\bf{r}}}}}}}^{{\prime} }}\cdot {{{{{{\bf{S}}}}}}}_{{{{{{\bf{r}}}}}}}\times {{{{{{\bf{S}}}}}}}_{{{{{{{\bf{r}}}}}}}^{{\prime} }}\right)-\mathop{\sum}\limits _{{{{{{\bf{r}}}}}}}{{{g}}}{{{{\mu }}}}_{{{{B}}}}{{{{B}}}}_{{{{z}}}}{{{{{{\bf{S}}}}}}}_{{{{{{\bf{r}}}}}}}\cdot \hat{{{{{{\bf{z}}}}}}}-\mathop{\sum}\limits_{{{{{{\bf{r}}}}}}}\\ 	 {{{{{\boldsymbol{+}}}}}}\,\frac{{{{{I}}}}_{{{{{{\mbox{dp}}}}}}}}{{{{2}}}}\mathop{\sum}\limits_{{{{{{\bf{r}}}}}}\ne{{{{{{\bf{r}}}}}}}^{{{{\prime} }}}}\left[\frac{{{{{{{\bf{S}}}}}}}_{{{{{{\bf{r}}}}}}} {{{\cdot }}}{{{{{{\bf{S}}}}}}}_{{{{{{{\bf{r}}}}}}}^{{{{\prime} }}}}}{{\left|{{{{{\bf{r}}}}}}-{{{{{{\bf{r}}}}}}}^{{{{\prime} }}}\right|}^{{{{3}}}}}-\frac{{{{3}}}\left\{{{{{{{\bf{S}}}}}}}_{{{{{{\bf{r}}}}}}}{{{\cdot }}}\left({{{{{\bf{r}}}}}}-{{{{{{\bf{r}}}}}}}^{{{{\prime} }}}\right)\right\}\left\{{{{{{{\bf{S}}}}}}}_{{{{{{{\bf{r}}}}}}}^{{{{\prime} }}}}{{{\cdot }}}\left({{{{{\bf{r}}}}}}-{{{{{{\bf{r}}}}}}}^{{{{\prime} }}}\right)\right\}}{{\left|{{{{{\bf{r}}}}}}-{{{{{{\bf{r}}}}}}}^{{{{\prime} }}}\right|}^{{{{5}}}}}\right]{{{,}}}$$

where **S**_**r**_ is a spin vector defined at **r** = (*x*, *y*, *z*) with lattice constant *a* = 1. The first two terms are nearest neighbor interactions, including the ferromagnetic exchange coupling ${J}_{{{{{{\bf{r}}}}}}{{{{{\boldsymbol{,}}}}}}{{{{{{\bf{r}}}}}}}^{{{{\prime} }}}}\,={J}\left({\delta }_{{{{{{\bf{r}}}}}}-{{{{{{\bf{r}}}}}}}^{{{{\prime} }}},\pm \hat{x}}+{\delta }_{{{{{{\bf{r}}}}}}-{{{{{{\bf{r}}}}}}}^{{{{\prime} }}},\pm \hat{y}}+{\delta }_{{{{{{\bf{r}}}}}}-{{{{{{\bf{r}}}}}}}^{{{{\prime} }}},\pm \hat{z}}\right)$ with *J* > 0 and the anisotropic Dzyaloshinskii–Moriya interaction ${{{{{{\bf{D}}}}}}}_{{{{{{\bf{r}}}}}}{{{{{\boldsymbol{,}}}}}}{{{{{{\bf{r}}}}}}}^{{{{\prime} }}}}\,={D}(\mp \hat{x}{\delta }_{{{{{{\bf{r}}}}}}-{{{{{{\bf{r}}}}}}}^{{{{\prime} }}},\pm \hat{x}}\pm \hat{y}\,{\delta }_{{{{{{\bf{r}}}}}}-{{{{{{\bf{r}}}}}}}^{{{{\prime} }}},\pm \hat{y}})$. The third term accounts for the external magnetic field ${B}_{z}\hat{z}$, where *g* and *μ*_*B*_ denote the g-factor and Bohr magneton, respectively. The model also includes easy-axis anisotropy (*K* > 0) and long-range dipolar interaction with coupling strength *I*_dp_. Energetically stable magnetic configurations are obtained by Monte Carlo simulated annealing.

Figure 5 in the main text shows the classical ground-state magnetic texture for 60 × 60 × 5 spins with free boundary conditions, $D/J=1.0,{g}{\mu }_{B}{B}_{z}/{JS}=0.3$, and *K* = *I*_dp_ = 0.0, at zero temperature. The magnetic texture was further relaxed employing the Landau–Lifshitz–Gilbert equation. A study of the magnetic configurations that result from the competition among the dipolar interaction, Dzyaloshinskii–Moriya interaction, and easy-axis anisotropy, at a finite temperature, can be found in the Fig. S2.

## Supplementary information

Supplementary Information

## Data Availability

The data that support the finding of this study are available from the corresponding author upon reasonable request.
