# Supplementary material for: Observation of fractional spin textures in a Heusler material
Source: Nat Commun. 2022 Apr 29;13:2348. doi: 10.1038/s41467-022-29991-1 (PMC9054820; doi:10.1038/s41467-022-29991-1)
Supplement: Supplementary file 1 — Supplementary Information [file 41467_2022_29991_MOESM1_ESM.pdf]

Supplementary Information

Observation of fractional spin textures in a Heusler material

Jagannath Jena[1], Börge Göbel[2], Tomoki Hirosawa[3,4], Sebastián A. Díaz[4,5], Daniel Wolf[6], Taichi Hinokihara[3,7], Vivek Kumar[8], Ingrid Mertig[2], Claudia Felser[8], Axel Lubk[6], Daniel Loss[4], and Stuart Parkin[1*]

[1] Max Planck Institute of Microstructure Physics, Weinberg 2, 06120 Halle, Germany
[2] Institute of Physics, Martin Luther University Halle-Wittenberg, 06120 Halle, Germany
[3] Department of Physics, University of Tokyo, Bunkyo, Tokyo 113-0033, Japan
[4] Department of Physics, University of Basel, Klingelberg Strasse 82, 4056 Basel, Switzerland
[5] Faculty of Physics, University of Duisburg-Essen, 47057 Duisburg, Germany
[6] Institute for Solid State Research, IFW Dresden, Helmholtzstrasse 20, 01069 Dresden, Germany
[7] Elements Strategy Initiative Center for Magnetic Materials, National Institute for Materials Science, Tsukuba, Ibaraki 305-0047, Japan
[8] Max Planck Institute for Chemical Physics of Solids, Nöthnitzer Strasse 40, 01187 Dresden, Germany

*Corresponding author : stuart.parkin@mpi-halle.mpg.de

Magnetic textures and competing interactions

Previous studies have pointed out that the dipolar interaction plays an important role in Heusler compounds such as $Mn_{1.4}Pt_{0.9}Pd_{0.1}Sn$[1,2]. This is particularly prominent at low temperatures, where elliptically deformed Bloch skyrmions are stabilized despite the anisotropic Dzyaloshinskii–Moriya interaction that favors antiskyrmions[1]. In our studies, at higher temperatures - room temperature and above - we do not observe these elliptically deformed Bloch skyrmions but rather only antiskyrmions as the concomitant decrease in the total magnetization of the sample suppresses the dipolar interactions. Therefore, this Heusler material provides a unique opportunity to study the competition between the Dzyaloshinskii–Moriya interaction, dipolar interactions, as well as the easy-axis anisotropy, at finite temperatures.

We have searched for parameters within our atomistic spin Hamiltonian model (see Methods) that can qualitatively reproduce the experimental results of the main text. Our system consists of 50 × 50 × 5 spins with free boundary conditions, $I_{dp} = 0.05$, and $g\mu_B B_z/JS = 0.05$. To efficiently perform Monte Carlo simulations, we employ the stochastic cut-off (SCO) method adapted for long-range dipolar interactions, which drastically reduces the computational cost for three-dimensional systems[3-5]. Our bond-switching algorithm for implementing the SCO method is based on Ref. 5. Starting from a thermal configuration, the temperature is lowered from $T/J = 20$ to $T/J = 0.7$ in 30 steps, each one consisting of 20,000 Monte Carlo steps. The magnetic configuration for each parameter set $\{D/J, K/J\}$ is obtained by taking the thermal average over 1000 samplings.

Fig. S2a-d show the magnetic textures obtained with increasing easy-axis anisotropy for $D/J = 0.07$. When the easy-axis anisotropy is sufficiently small, the in-plane vortex phase is the most stable configuration and carries no topological charge. We conclude that the easy-axis anisotropy of our $Mn_{1.4}Pt_{0.9}Pd_{0.1}Sn$ samples must be sufficiently large since no in-plane vortices were revealed by LTEM imaging. As the easy-axis anisotropy is increased, various topological objects are stabilized. Crucially, elliptical Bloch skyrmions are first stabilized at $K/J = 0.35$, which have a swirling spin texture like the vortex phase as shown in Fig. S2b. Further increasing the easy-axis anisotropy favors the formation of antiskyrmions. Non-topological bubbles are obtained as a metastable state at the boundary between antiskyrmions and elliptical Bloch skyrmions. Therefore, our study suggests that the easy-axis anisotropy also plays an important role in stabilizing elliptical Bloch skyrmions in Heusler materials.

In Fig. S2e we summarize our results on the stability of different topological spin structures as a function of the Dzyaloshinskii–Moriya interaction D and easy-axis anisotropy

K, obtained by single-shot Monte Carlo simulations. We conclude that the stability of elliptical Bloch skyrmions and antiskyrmions is determined by the competition among the dipolar interaction, Dzyaloshinskii–Moriya interaction, and easy-axis anisotropy. In particular, for $D/J < 0.08$, Bloch skyrmions could coexist with antiskyrmions depending on the strength of the easy-axis anisotropy. Since the demagnetization field becomes larger at low temperatures due to an increased saturation magnetization, elliptical Bloch skyrmions become more favourable than antiskyrmions at low temperatures in Heusler materials.

Stability of fractional (anti)skyrmions

As discussed in the main text, the ferromagnetic phase in chiral magnets becomes unstable against nucleation of helical domains at the edges of the sample below the critical magnetic field. To confirm the stability of edge magnetic textures in systems with $D_{2\mathrm{d}}$ symmetry and dipolar interaction, we studied the energy landscape of fractional skyrmions as a function of the applied magnetic field fixing the other magnetic parameters at $\{D/J, K/J, I_{dp}\} = \{0.05, 0.45, 0.05\}$. Using the same protocol of efficient Monte Carlo simulations as in the previous section, we prepared the spin configuration of an antiskyrmion, an elliptical Bloch skyrmion, a fractional antiskyrmion, and a fractional Bloch skyrmion in a 50 × 50 × 5 spin system with free boundary conditions at $T/J = 0.7$. In order to evaluate the energy of each metastable configuration, the temperature was abruptly dropped to zero and then each object was relaxed employing the Landau-Lifshitz-Gilbert equation for 2,000 time steps. The energy was computed as a function of magnetic field by slowly changing the magnetic field over 2,000 time steps and undergoing further relaxation for another 2,000 time steps.

Fig. S3 shows the energy of each magnetic texture at various magnetic fields in comparison to the ferromagnetic phase. We find that both fractional antiskyrmions and fractional Bloch skyrmions become more stable than antiskyrmions at approximately $g\mu_B B_z/JS = 0.01$. These results are consistent with the experimental observation of stable fractional skyrmions at low magnetic fields in Heusler materials. For these parameters, the energy of elliptical Bloch skyrmions is slightly lower than antiskyrmions at $g\mu_B B_z/JS > 0.01$. We should note that the stability between antiskyrmions and elliptical Bloch skyrmions depends on the relative strength of D/J and K/J in comparison to the value of I_{dp}. In fact, we find that antiskyrmions become energetically more favourable than elliptical Bloch skyrmions at $D/J = 0.07$ and $K/J = 0.45$ for all magnetic fields.

Dynamics of conversion, annihilation, and merging

To further investigate the conversion and annihilation mechanisms as the magnetic field is varied, we performed atomistic spin dynamics simulations. An initial spin configuration was prepared for 200 × 200 × 5 spins with free boundary conditions using the efficient Monte Carlo simulated annealing protocol described in the previous section. We chose magnetic parameters, $\{D/J, K/J, g\mu_B B_z/JS, I_{dp}\} = \{0.05, 0.45, 0.06, 0.05\}$, that allow the formation of a mixture of antiskyrmions, elliptical Bloch skyrmions, and non-topological bubbles. Then, as the magnetic field was varied, the initial spin configuration was time-evolved using the stochastic Landau-Lifshitz-Gilbert equation[6] with the temperature and Gilbert damping fixed at $T/J = 0.7$ and $\alpha = 0.3$, respectively.

The magnetic field protocol begins with a linear reduction from $g\mu_B B_z/JS = 0.06$ to $g\mu_B B_z/JS = -0.015$ by 10 decrements of 200 time steps each. During the next 8,000 time steps the magnetic field is kept constant allowing the magnetic texture to relax. Then the field is linearly increased by 10 increments of 200 time steps from $g\mu_B B_z/JS = -0.015$ to $g\mu_B B_z/JS = 0.065$ and is kept at that final value for 18,000 more time steps. Each time step has a duration of $\hbar/JS$, which for $J = 1$ meV and $S = 1$ approximates to $\hbar/JS \approx 0.7$ ps.

Supplementary Video 1 shows the time evolution of the magnetic texture obtained by taking the thermal average over consecutive 200 time steps intervals. As the magnetic field is lowered, stripe domains are formed in the interior via elongation or merging of objects. We find that the merging, mediated by the formation of monopoles[7], often occurs between different topological objects having parallel in-plane magnetizations at the merging points. After the field reaches the minimum value $g\mu_B B_z/JS = -0.015$, more objects in the interior merge resulting in longer stripe domains, and fractional objects along the sample edges are formed. Here, we note that thermal fluctuations are necessary to overcome the energy barrier induced by the texture edge twist. The importance of thermal fluctuations becomes more evident as the magnetic field is increased again. While the annihilation and conversion occur spontaneously for smaller fractional objects, the conversion of a long fractional object with a longer extension into the bulk region to an integer topological charged object requires a long relaxation time. The total number of interior objects got reduced because their initial merging formed stripe domains that then simply shrank into single objects. Our simulation successfully reproduces the experimentally observed conversion and annihilation mechanisms, and suggests the importance of thermal fluctuations for the continuous transformation of topological charges.

We also studied the merging of fractional antiskyrmions under negative magnetic fields. The parameters were chosen as $\{D/J, K/J, g\mu_B B_z/JS, I_{dp}\} = \{0.07, 0.5, 0.06, 0.05\}$, where antiskyrmions become more stable than elliptical Bloch skyrmions. Indeed, the initial configuration obtained by the efficient Monte Carlo simulated annealing protocol consists of square-shaped antiskyrmions for 200 × 200 × 5 spins with free boundary conditions. The atomistic spin dynamics simulation was performed analogously to the above calculation, reducing the magnetic field from $g\mu_B B_z/JS = 0.06$ to $g\mu_B B_z/JS = -0.045$, to $g\mu_B B_z/JS = -0.06$, and finally to $g\mu_B B_z/JS = -0.075$ with 2,000 time steps for reduction followed by 8,000 times steps for relaxation in each step. The result is shown in Supplementary Video 2.

As the magnetic field is decreased from $g\mu_B B_z/JS = 0.06$ to $g\mu_B B_z/JS = -0.045$, stripe domains are formed via elongation and merging of antiskyrmions. It should be noted that only one fractional antiskyrmion is formed at this magnetic field, indicating a larger energy barrier to overcome than in Supplementary Video 1. Further reduction in the magnetic field to $g\mu_B B_z/JS = -0.06$ results in transforming the majority of antiskyrmions near the sample edges into fractional antiskyrmions. When the magnetic field is lowered to $g\mu_B B_z/JS = -0.075$, fractional antiskyrmions merge by first touching at one point at either the interior or at the edge of the sample, which then zips them together resulting in a field polarized region in the vicinity of the edge. Since fractional antiskyrmions carry approximately a half quantized topological charge, their merging process implies the emergence of fractionally charged monopoles[7]. An in-depth discussion of fractionally charged monopoles will be provided elsewhere.

Connection between real- and reciprocal-space topologies in (anti)skyrmion crystals

Recently, it has been shown that (anti)skyrmion crystals support a higher-order topological phase characterized by a nontrivial magnonic quadrupole moment[9], protected by crystalline symmetries and whose hallmark signatures are robust magnonic corner states. Using nested Wilson loops adapted to magnonic systems, this novel topological phase was found to exist for a broad range of magnetic fields. However, in order to realize these magnonic corner states, it is essential to preserve the protecting symmetries at the sample boundaries. This is particularly important in magnetic systems, as the magnetization profile near the edges of the sample could be easily distorted from that of the magnetic unit cell in the bulk of a sample.

A crucial finding in Ref. 9 is that the classical spin ground state in a finite sample exhibits a reconstruction with fundamental consequences for the magnonic topological

excitations. As discussed in the main text, the twisted spin configuration along the sample edges facilitates the formation of fractional (anti)skyrmions below the critical magnetic field. The mutual repulsion between fractional (anti)skyrmions and bulk (anti)skyrmions results in their self-assembly that restores the symmetries protecting the bulk topology in reciprocal space. As an important consequence, the topological behaviour of the magnon states in the bulk gives rise to special magnon edge states at the sample boundaries. This is a manifestation of the celebrated bulk-boundary correspondence. Without fractional (anti)skyrmions there would be no such edge states and the bulk-boundary correspondence would not hold. In particular, for the system under consideration in-gap magnon states with large probability amplitude are predicted to emerge at the boundary, more precisely, at each corner of the sample. In other words, fractional (anti)skyrmions are a necessary condition for the existence of such magnon corner states. In contrast, above the critical magnetic field where fractional (anti)skyrmions are no longer stable these magnonic in-gap states are buried among bulk modes, and spread over the edges and into the bulk of the sample. Therefore, the observation of fractional (anti)skyrmions in Heusler materials constitutes an important steppingstone towards the direct experimental observation of the magnonic corner states. For interested readers, we refer to Ref. 9 for further details.

Formation of fractional antiskyrmions at low temperature

In our $Mn_{1.4}Pt_{0.9}Pd_{0.1}Sn$ sample, we find that antiskyrmions are stable at room temperature and above, whereas elliptical Bloch skyrmions are found at low temperatures[1]. However, one is able to observe antiskyrmions at low temperatures using the following field cooling procedure, as schematically shown in Fig. S5a. On stabilizing a square antiskyrmion lattice at room temperature in the presence of a constant perpendicular magnetic field, we reduce the specimen's temperature to 100 K. Fig. S5b shows a typical Lorentz transmission electron microscopy (LTEM) image taken at 100 K and in the presence of a 200 mT field. Here, we focus on the region at the edge of the sample identified by the yellow rectangle in Fig.S5b to illustrate the formation of fractional antiskyrmions. The initial state within this rectangle consists of square antiskyrmions as shown in Fig. S5c. On reducing the magnetic field, those antiskyrmions that are touching the edge of the sample become fractional antiskyrmions (Fig. S5d). The size of these fractional nano-objects reduces as the field crosses zero and increases in strength in the negative direction (Fig. S5e and S5f). The fractional antiskyrmions become triangularly-shaped in the field region from -72 mT to -224 mT (Fig. S5g and S5j).

Supplementary figures

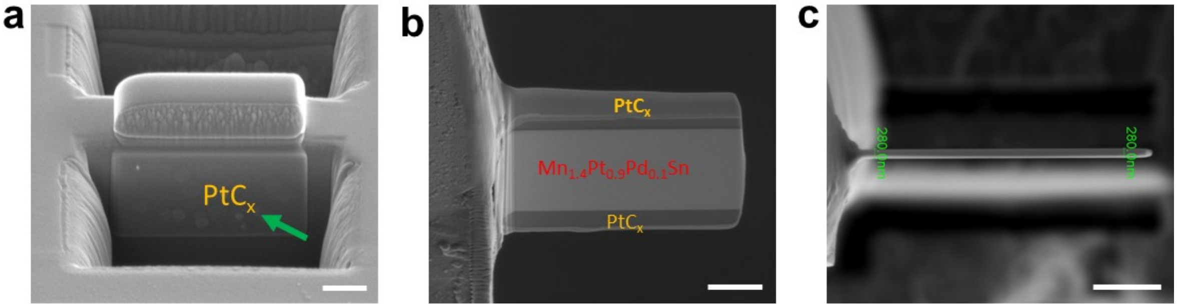

Fig. S1. **Lamella preparation using focused ion beam milling.** Lamellae specimens used for LTEM imaging are formed from bulk polycrystalline samples of $Mn_{1.4}Pt_{0.9}Pd_{0.1}Sn$ using focused ion milling. An individual grain, that is single crystalline, is identified whose crystal orientation is such that the [001] axis is perpendicular to the lamella that will be formed. EBSD (electron back scattering diffraction) is used for this purpose within the focused ion beam (FIB) apparatus. (**a**) Before lifting out the specimen from the bulk sample, the surfaces of the two sides of the specimen through which the electron beam is transmitted are polished using a low energy Ga^+ ion beam of 2 keV and 4 pA until the surfaces are smooth. Then, PtC_x is deposited on both these surfaces using an electron beam to decompose a Pt organometallic compound. One of these surfaces is indicated by the green arrow. (**b**) Overview of the prepared lamella after removal from the host grain and attachment to a standard TEM copper grid. The deposited PtC_x layers on the top and bottom of the $Mn_{1.4}Pt_{0.9}Pd_{0.1}Sn$ lamella are indicated in the figure. (**c**) Shows the thickness of the prepared lamella at zero-tilt stage conditions. The lamella is ~280 nm thick. The white scale bars in each figure correspond to 2 μm. More details on the preparation method can be found elsewhere[1].

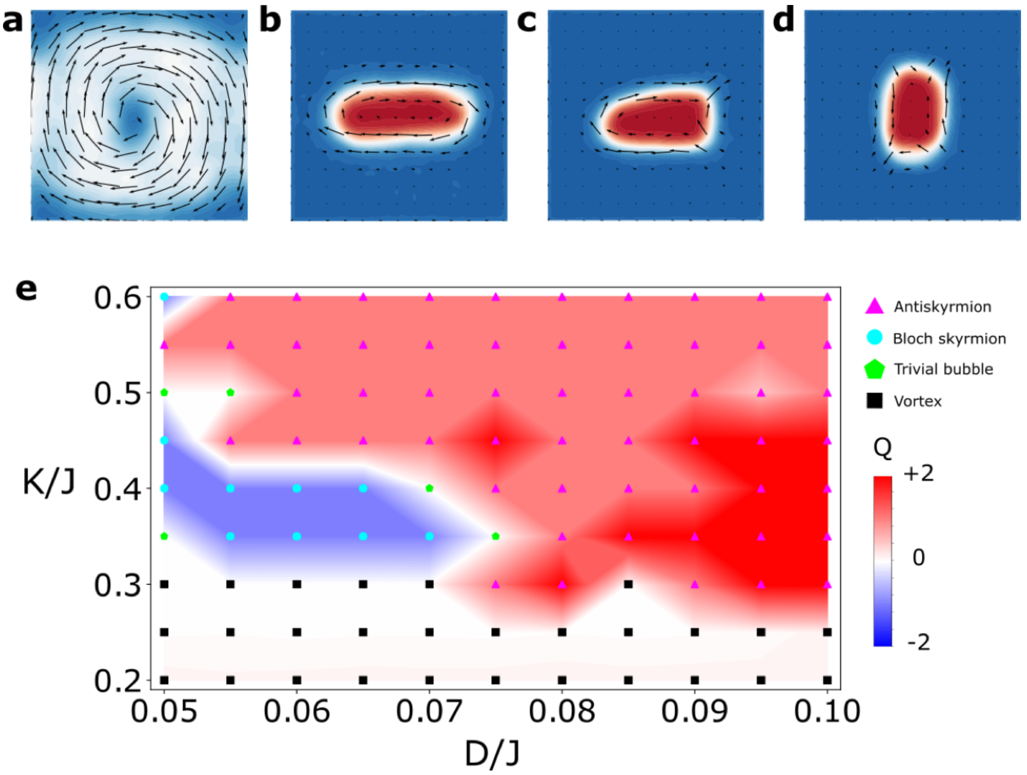

Fig. S2. Magnetic textures stabilized from the competition among the dipolar interaction, Dzyaloshinskii–Moriya interaction, and easy-axis anisotropy. (**a**)-(**d**) The magnetic textures of (**a**) a vortex, (**b**) a Bloch skyrmion, (**c**) a non-topological bubble, and (**d**) an antiskyrmion, stabilized at $D/J = 0.07$ with increasing easy-axis anisotropy ($K/J = 0.2, 0.35, 0.4, 0.45$). (**e**) Phase diagram as a function of the Dzyaloshinskii–Moriya interaction D and easy-axis anisotropy K. The color map indicates the net topological charge of the sample $Q = \sum_r \rho(\boldsymbol{r})$, where the topological charge density $\rho(\boldsymbol{r})$ for a discrete lattice is defined in Ref. 8. In some cases, we obtain $Q = 2$ for two antiskyrmions and $Q = 1/2$ for edge fractional antiskyrmions. All the results are obtained in a 50 × 50 × 5 spin system using Monte Carlo simulated annealing with $I_{dp} = 0.05$, $g\mu_B B_z/JS = 0.05$, and $T/J = 0.7$.

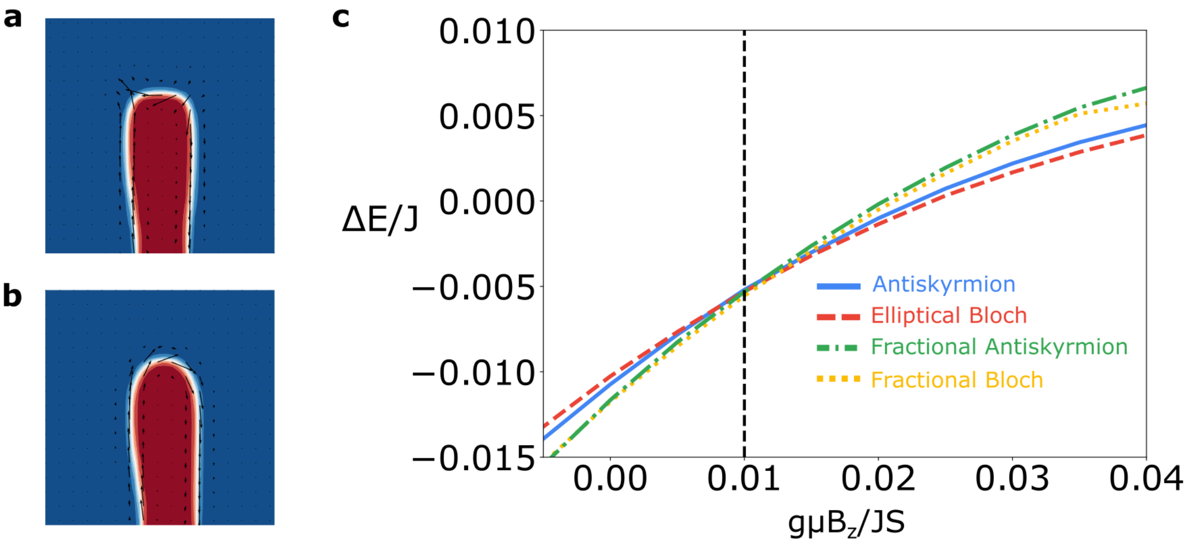

Fig. S3. Energy landscape of fractional skyrmions and antiskyrmions. (**a**)-(**b**) The magnetic textures of (**a**) a fractional antiskyrmion and (**b**) a fractional Bloch skyrmion relaxed at zero temperature and $g\mu_B B_z/JS = 0.03$. (**c**) Energy per site of antiskyrmions (solid blue), elliptical Bloch skyrmions (dashed red), fractional antiskyrmions (dash-dotted green), and fractional Bloch skyrmions (dotted yellow) at various magnetic fields in comparison to the ferromagnetic phase, defined as $\Delta E = \frac{E-E_{FM}}{N_{site}}$. The vertical dashed line indicates the critical magnetic field where fractional (anti)skyrmions become more stable than (anti)skyrmions. Magnetic parameters read $\{D/J, K/J, I_{dp}\} = \{0.05, 0.45, 0.05\}$.

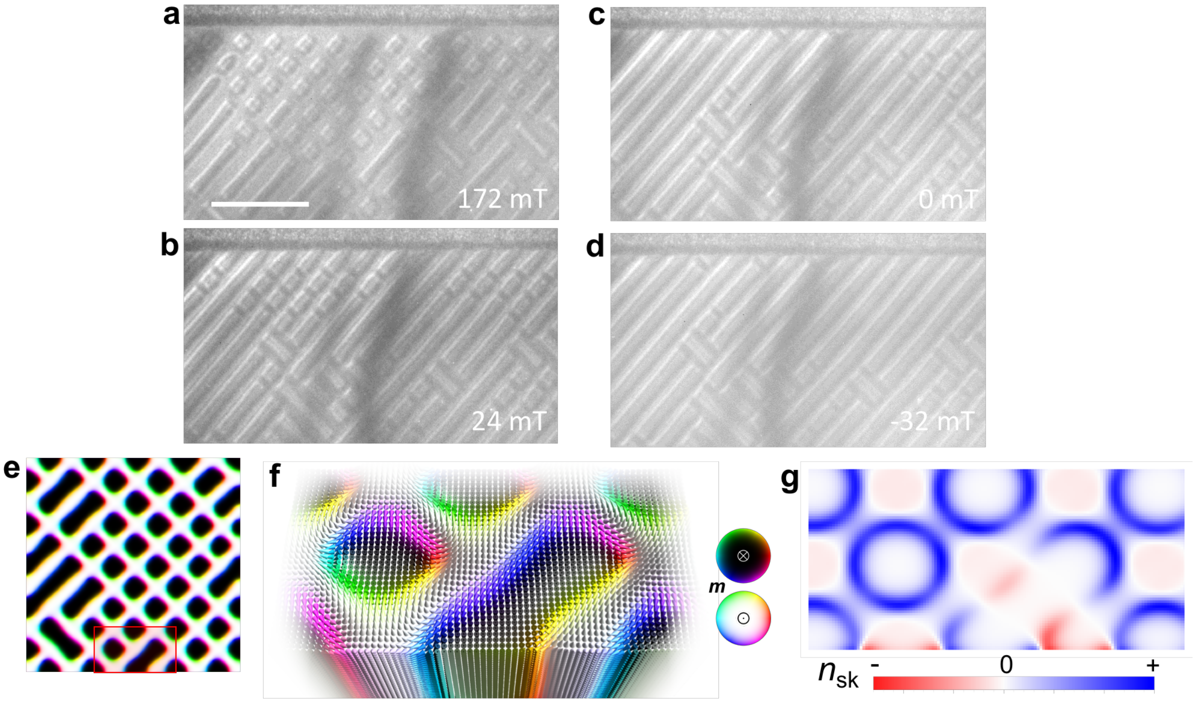

Fig. S4. Formation of fractional antiskyrmions from a disordered state. (**a**) The lamella is filled with a less dense lattice of antiskyrmions and short helices (rectangular shape) at room temperature and 172 mT. (**b**) At 24 mT, antiskyrmions near the edge get elongated and fractional antiskyrmions form. (**c, d**) At 0 and -32 mT, fractional antiskyrmions at the edge join with interior antiskyrmions and the region is filled with long helices. (**e**) Micromagnetic simulation of the disordered antiskyrmion lattice with fractional antiskyrmions at the edge. The color code corresponds to the orientation of the magnetic moments. **(f)** A magnified view of the area identified by the red rectangle in (**e**) with arrows indicating the orientation of the magnetic moments. **(g)** The topological charge density of the spin texture in (**f**). The scale bar in (**a**) corresponds to 1 µm.

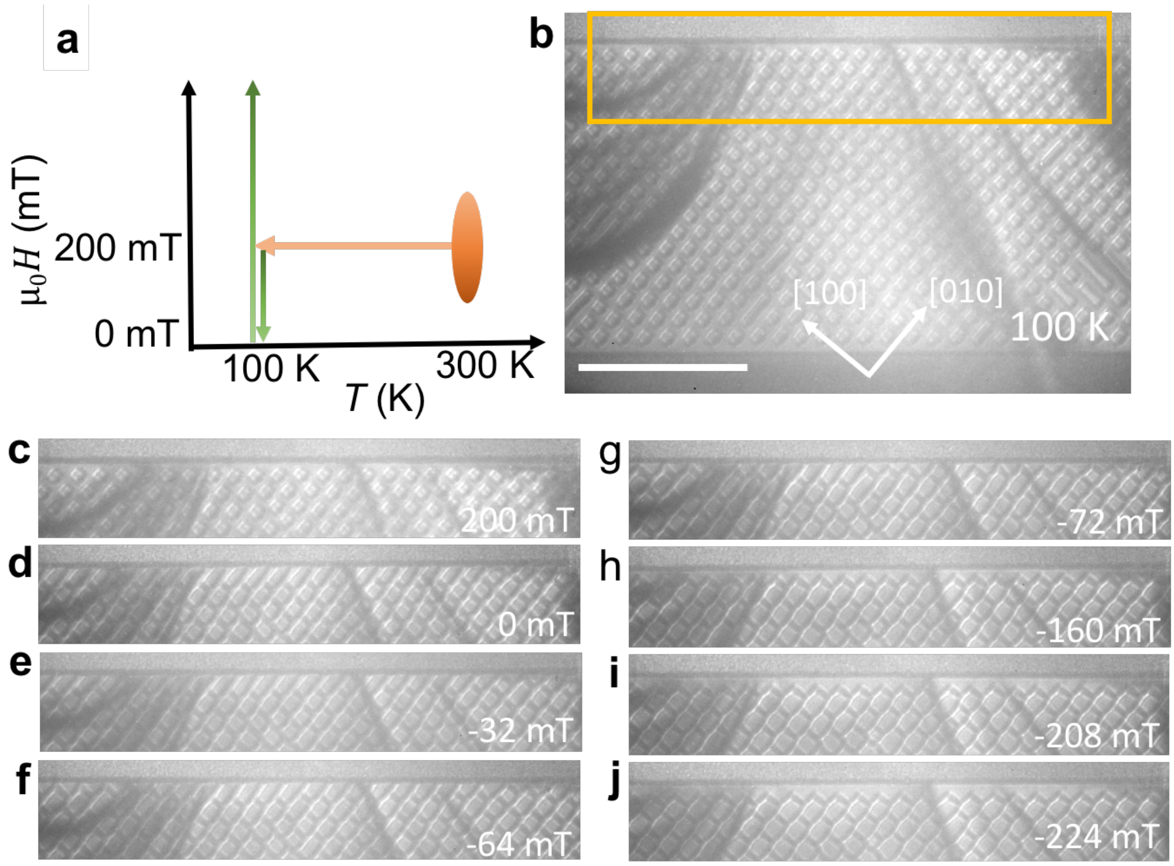

Fig. S5. Fractional antiskyrmions at low temperature. (**a**) Schematic of field cooling protocol to stabilize a metastable antiskyrmion lattice at 100 K. (**b**) Square antiskyrmion lattice state at 100 K after cooling the specimen from 300 K in a field of 200 mT. The region marked by the yellow rectangle in (**b**) is shown in (**c-i**). Fractional antiskyrmions are formed at the $Mn_{1.4}Pt_{0.9}Pd_{0.1}Sn$ - PtC_x interface on reducing the magnetic field. The scale bar in (**b**) corresponds to 2 µm.

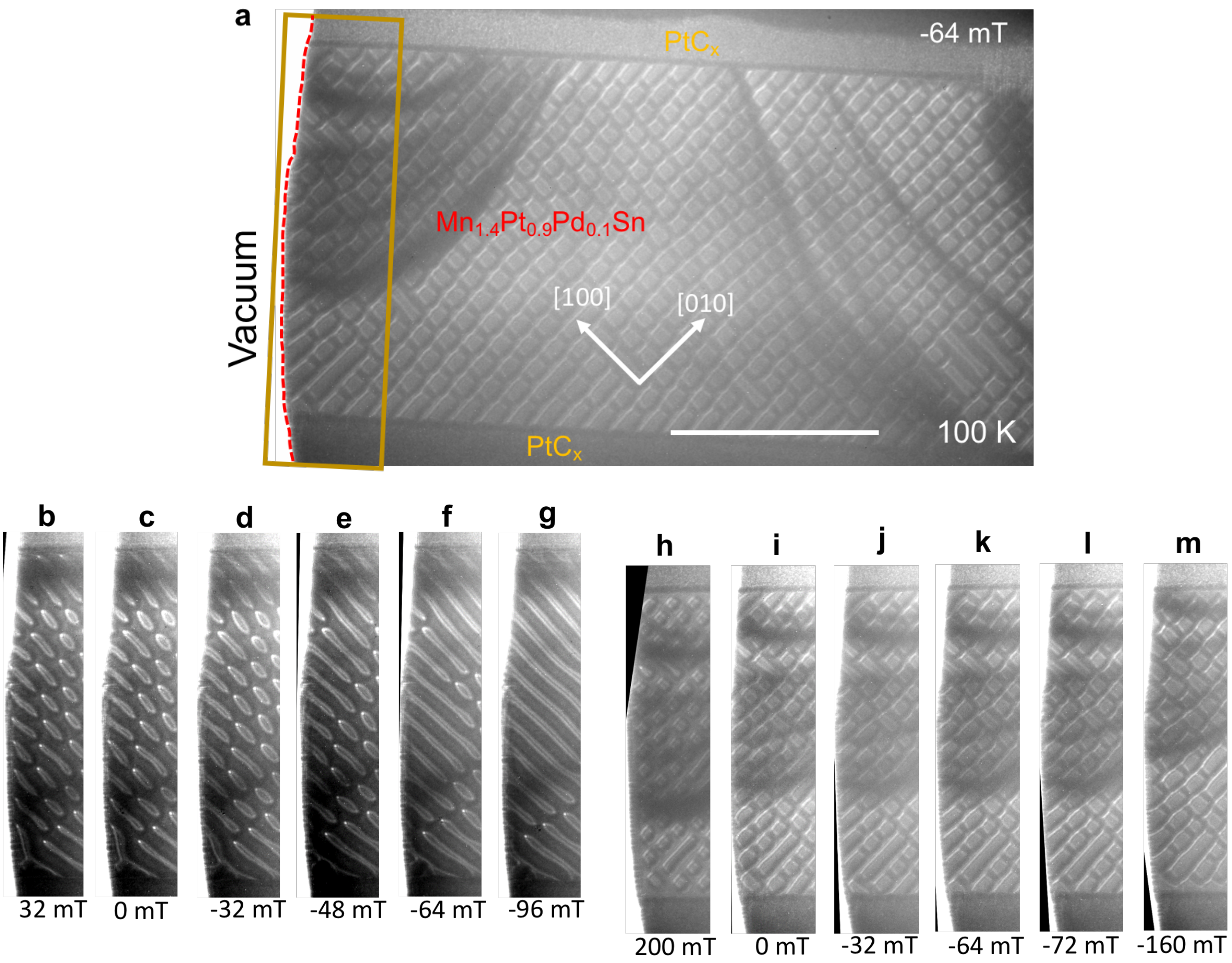

Fig. S6. Fractional nano-objects at the interface of $Mn_{1.4}Pt_{0.9}Pd_{0.1}Sn$ and vacuum. (**a**) An extended lamella formed from $Mn_{1.4}Pt_{0.9}Pd_{0.1}Sn$ showing three interface regions: top and bottom interfaces are formed when PtC_x is deposited on the sides of the lamella in the FIB system and the third interface is formed at the interface of the lamella and vacuum. This last interface is marked by the red dashed curve. The region identified by the yellow rectangle is shown at different fields and various protocols in (**b-g**) and (**h-m**) at 100 K. In (**b-g**) the sample was cooled in zero field from room temperature to 100 K. Then, at 100 K, the field is systematically reduced to observe fractional Bloch skyrmions after a dense lattice state of elliptical Bloch skyrmions is formed at higher magnetic fields. In (**h-m**) the sample is field cooled in 200 mT from 300K to 100 K. Then the magnetic field is reduced to observe the fractional antiskyrmions. The scale bar in (**a**) corresponds to 2 µm.

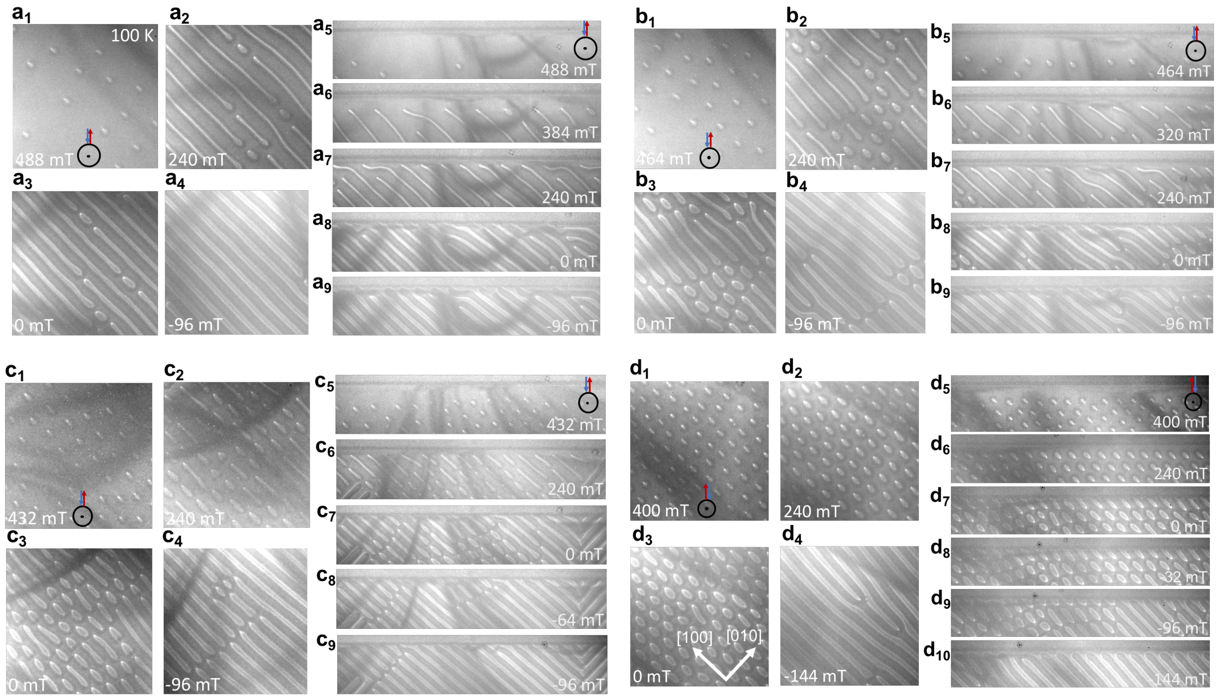

Fig. S7. Density dependent formation of fractional nano-objects: (**a_1, b_1, c_1, d_1**) Nano-objects with various densities are formed at different strengths of the magnetic field after the temporary application of an in-plane field component via tilting of the sample. The temporary tilting angle is ~40°. The various states are shown at 488 mT, 464 mT, 432 mT and 400 mT, respectively. The blue and red arrows indicate the direction of the temporary tilting of the specimen. (**a_2-a_4, b_2-b_4, c_2-c_4, d_2-d_4**) After that, the field is reduced till the formation of a helical state. LTEM images are shown from, firstly, a typical region of the lamella away from the edge. This region is square shaped. Secondly, the same yellow rectangular region identified in Fig. S4, that is representative of the interface between $Mn_{1.4}Pt_{0.9}Pd_{0.1}Sn$ and PtC_X. (**a_5-a_9, b_5-b_9, c_5-c_9, d_5-d_{10}**) The interior and interface regions are shown at various magnetic fields. The number of fractional nano-objects formed at the interface depends on the density of the nano-objects in the lamella. The higher the density of these objects, the larger is the number of fractional objects at the interface. The dimension of the square shaped interior region is 2500 nm × 2500 nm and the interface region has dimensions of 1300 nm × 6000 nm.

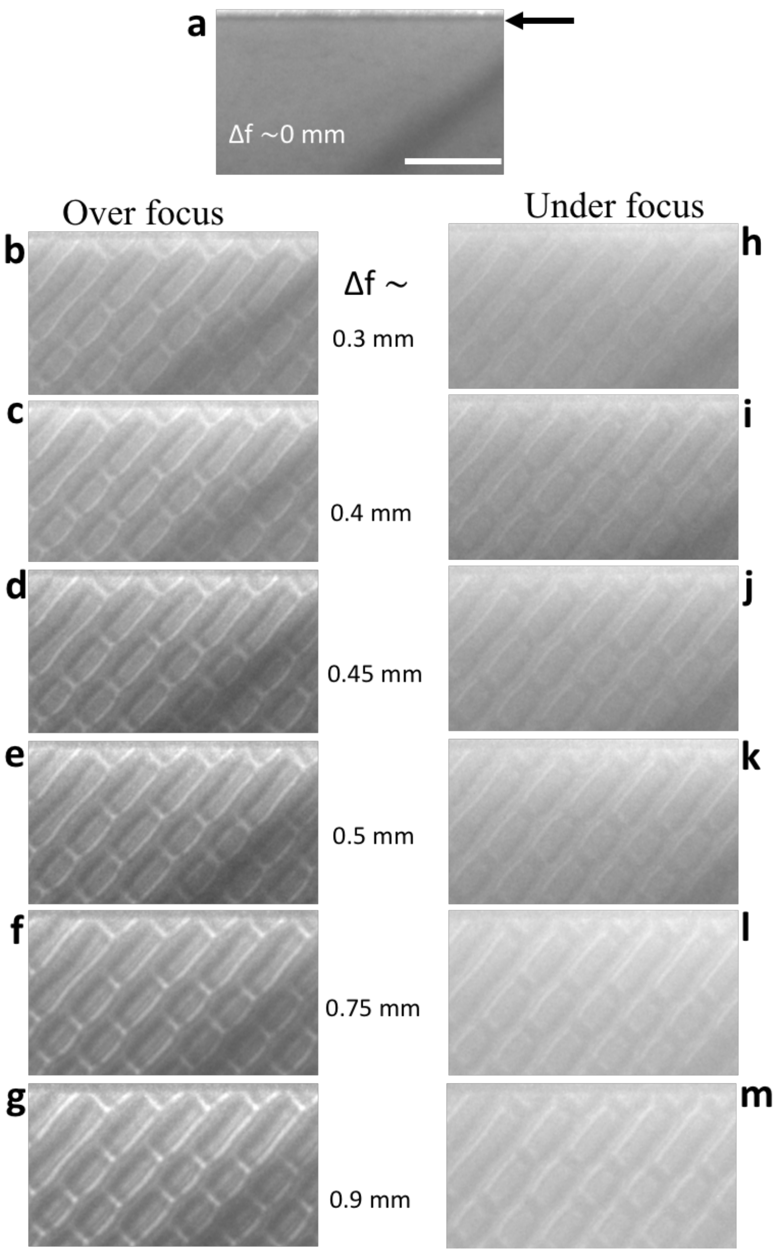

Fig. S8. Fractional antiskyrmions at different defocus values. (a) At in-focus condition (Δf ~ 0 mm), the specimen does not show magnetic contrast. The boundary between $Mn_{1.4}Pt_{0.9}Pd_{0.1}Sn$ and PtC_x is marked by a black arrow. **(b-g)** LTEM contrasts at over-focus values of ~0.3, 0.4, 0.45, 0.5, 0.75, 0.9 mm, respectively. **(h-m)** LTEM contrasts at under-focus values of ~0.3, 0.4, 0.45, 0.5, 0.75, 0.9 mm, respectively. The images are taken at -14.5 mT and 200 K. Fractional antiskyrmions at the boundary are stabilized using the same protocol as used in Fig. S4. The scale bar in (a) corresponds to 300 nm.

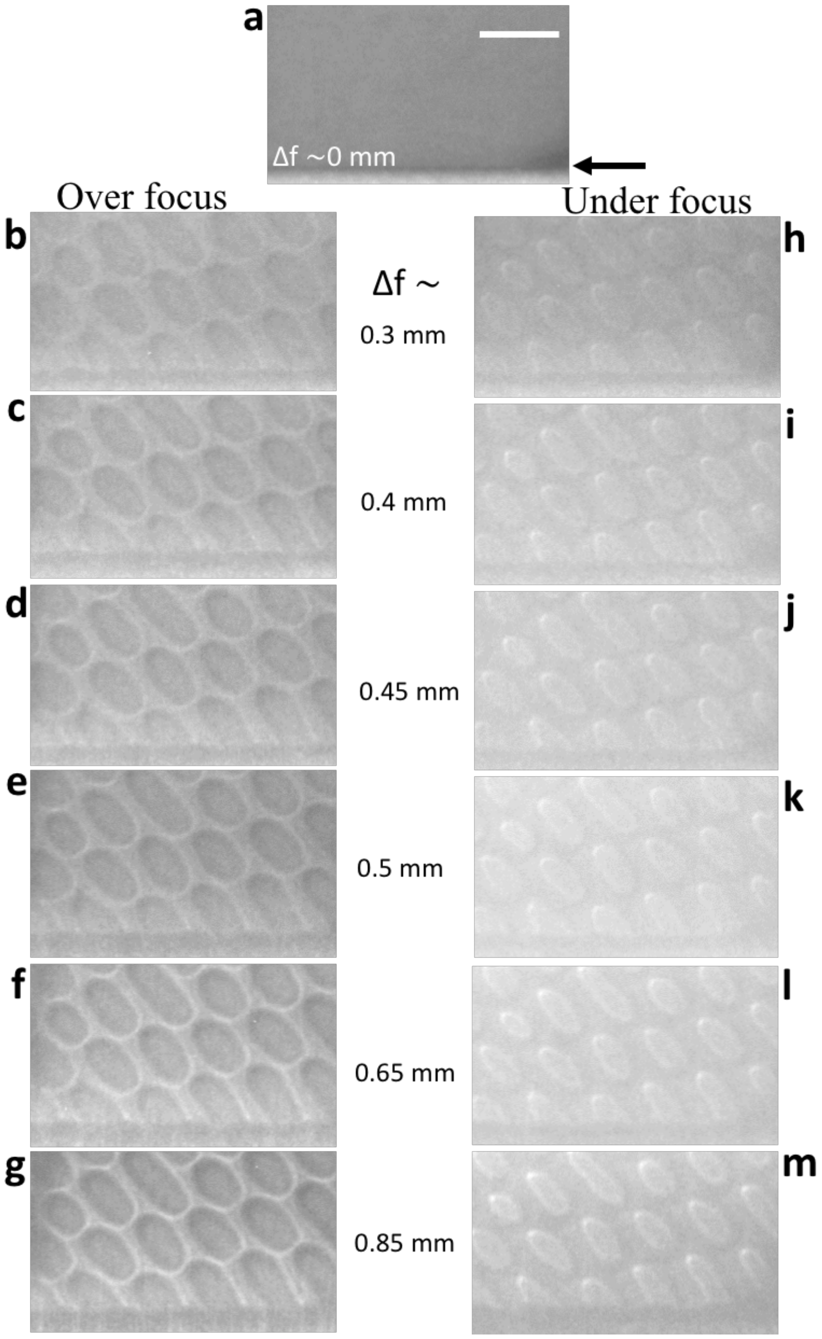

Fig. S9. Fractional Bloch skyrmions at different defocus values. (a) At in-focus condition ($\Delta f \sim 0$ mm), the specimen does not show magnetic contrast. The boundary between $Mn_{1.4}Pt_{0.9}Pd_{0.1}Sn$ and PtC_x is marked by a black arrow. **(b-g)** LTEM contrasts at over-focus values of ~0.3, 0.4, 0.45, 0.5, 0.65, 0.85 mm, respectively. **(h-m)** LTEM contrasts at under-focus values of ~0.3, 0.4, 0.45, 0.5, 0.65, 0.85 mm, respectively. The images are taken at -16 mT and 125 K. Fractional Bloch skyrmions are stabilized using the same protocol as used in Fig. 2. The scale bar in (a) corresponds to 300 nm.

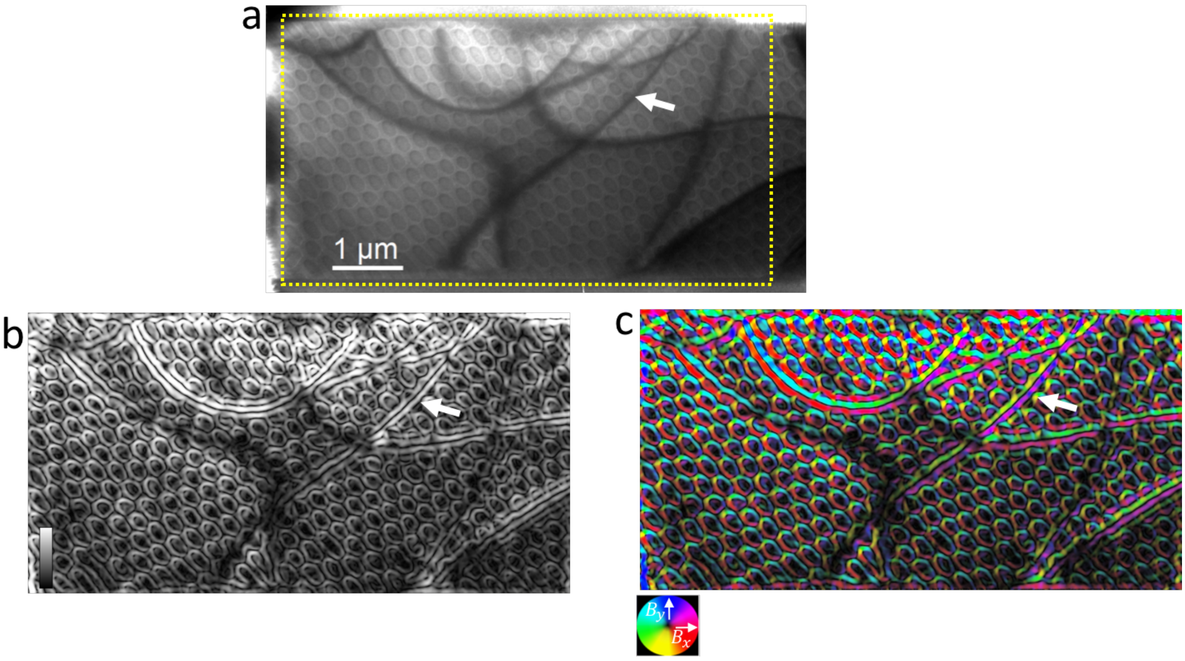

Fig. S10: In-plane magnetic induction map obtained by transport of intensity equation (TIE) analysis. (a) Experimental LTEM image of an extended lamella at -16 mT and 125 K exhibits elliptical Bloch skyrmions. The image is taken at an over-focus value of ~0.3 mm. **(b, c)** ***B***-field magnitude (b) and orientation (c) of the magnetic nano-textures reconstructed using TIE analysis taking into account a lamella thickness of 280 nm. Grey color scale in (b) ranges from 0 to 72 mT. The color wheel in (c) represents in-plane components of the magnetic induction ***B***. The black lines (one of which is marked by a white arrow) in (a) correspond to bending contours in the lamella, giving rise to artificial (non-magnetic) features in the TIE analysis as shown in (b) and (c).

References

1 Jena, J. *et al.* Elliptical Bloch skyrmion chiral twins in an antiskyrmion system. *Nat. Commun.* **11**, 1115 (2020).

2 Peng, L. *et al.* Controlled transformation of skyrmions and antiskyrmions in a non-centrosymmetric magnet. *Nat. Nanotechnol.* **15**, 181-186 (2020).

3 Mak, C. Stochastic potential switching algorithm for Monte Carlo simulations of complex systems. *J. Chem. Phys.* **122**, 214110 (2005).

4 Sasaki, M. & Matsubara, F. Stochastic cutoff method for long-range interacting systems. *J. Phys. Soc. Jpn.* **77**, 024004 (2008).

5 Hinokihara, T., Nishino, M., Toga, Y. & Miyashita, S. Exploration of the effects of dipole-dipole interactions in $Nd_2Fe_{14}B$ thin films based on a stochastic cutoff method with a novel efficient algorithm. *Phys. Rev. B* **97**, 104427 (2018).

6 Skubic, B., Hellsvik, J., Nordström, L. & Eriksson, O. A method for atomistic spin dynamics simulations: implementation and examples. *J. Phys.: Condens. Matter* **20**, 315203 (2008).

7 Milde, P. *et al.* Unwinding of a skyrmion lattice by magnetic monopoles. *Science* **340**, 1076-1080 (2013).

8 Berg, B. & Lüscher, M. Definition and statistical distributions of a topological number in the lattice O (3) σ-model. *Nucl. Phys. B* **190**, 412-424 (1981).

9 Hirosawa, T., Díaz, S. A., Klinovaja, J. & Loss, D. Magnonic Quadrupole Topological Insulator in Antiskyrmion Crystals. *Phys. Rev. Lett.* **125**, 207204 (2020).
